# Invasiveness as a putative additional virulence mechanism of some atypical Enteropathogenic *Escherichia coli *strains with different uncommon intimin types

**DOI:** 10.1186/1471-2180-9-146

**Published:** 2009-07-21

**Authors:** Denise Yamamoto, Rodrigo T Hernandes, Miguel Blanco, Lilo Greune, M Alexander Schmidt, Sylvia M Carneiro, Ghizlane Dahbi, Jesús E Blanco, Azucena Mora, Jorge Blanco, Tânia AT Gomes

**Affiliations:** 1 Universidade Federal de São Paulo (UNIFESP), Rua Botucatu, 862, 3° andar, Vila Clementino, São Paulo, CEP 04023-062, Brazil; 2 Institut für Infektiologie, Zentrum für Molekularbiologie der Entzündung, Westfälische Wilhelms-Universität, Münster, Germany; 3 E. coli Reference Laboratory (LREC), Department of Microbiology and Parasitology, Faculty of Veterinary, University of Santiago de Compostela, Lugo, Spain; 4 Laboratório de Biologia Celular do Instituto Butantan, São Paulo, Brazil

## Abstract

**Background:**

Enteropathogenic *Escherichia coli *(EPEC) produce attaching/effacing (A/E) lesions on eukaryotic cells mediated by the outer membrane adhesin intimin. EPEC are sub-grouped into typical (tEPEC) and atypical (aEPEC). We have recently demonstrated that aEPEC strain 1551-2 (serotype O non-typable, non-motile) invades HeLa cells by a process dependent on the expression of intimin sub-type omicron. In this study, we evaluated whether aEPEC strains expressing other intimin sub-types are also invasive using the quantitative gentamicin protection assay. We also evaluated whether aEPEC invade differentiated intestinal T84 cells.

**Results:**

Five of six strains invaded HeLa and T84 cells in a range of 13.3%–20.9% and 5.8%–17.8%, respectively, of the total cell-associated bacteria. The strains studied were significantly more invasive than prototype tEPEC strain E2348/69 (1.4% and 0.5% in HeLa and T84 cells, respectively). Invasiveness was confirmed by transmission electron microscopy. We also showed that invasion of HeLa cells by aEPEC 1551-2 depended on actin filaments, but not on microtubules. In addition, disruption of tight junctions enhanced its invasion efficiency in T84 cells, suggesting preferential invasion via a non-differentiated surface.

**Conclusion:**

Some aEPEC strains may invade intestinal cells *in vitro *with varying efficiencies and independently of the intimin sub-type.

## Background

Enteropathogenic *Escherichia coli *(EPEC) are important human intestinal pathogens. This pathotype is sub-grouped into typical (tEPEC) and atypical (aEPEC) EPEC [1-3]. These sub-groups differ according to the presence of the EAF plasmid, which is found only in the former group [1,3]. Recent epidemiological studies have shown an increasing prevalence of aEPEC in both developed and developing countries [4-9].

The main characteristic of EPEC's pathogenicity is the development of a histopathologic phenotype in infected eukaryotic cells known as attaching/effacing (A/E) lesion. This lesion is also formed by enterohemorrhagic *E. coli *(EHEC), another diarrheagenic *E. coli *pathotype whose main pathogenic mechanism is the production of Shiga toxin [10]. The A/E lesion comprises microvillus destruction and intimate bacterial adherence to enterocyte membranes, supported by a pedestal rich in actin and other cytoskeleton components [11]. The ability to produce pedestals can be identified *in vitro* by the fluorescence actin staining (FAS) assay that detects actin accumulation underneath adherent bacteria indicative of pedestal generation [12]. The genes involved in the establishment of A/E lesions are located in a chromosomal pathogenicity island named the *l*ocus of *e*nterocyte *e*ffacement (LEE) [13]. These genes encode a group of proteins involved in the formation of a type III secretion system (T3SS), an outer membrane adhesin called intimin [14], its translocated receptor (*t*ranslocated *i*ntimin *r*eceptor, Tir), chaperones and several other effector proteins that are injected into the targeted eukaryotic cell by the T3SS [15,16].

Differentiation of intimin alleles represents an important tool for EPEC and EHEC typing in routine diagnosis as well as in pathogenesis, epidemiological, clonal and immunological studies. The intimin C-terminal end is responsible for receptor binding, and it has been suggested that different intimins may be responsible for different host tissue cell tropism (reviewed in [17]). The 5' regions of *eae *genes are conserved, whereas the 3' regions are heterogeneous. Thus far 27 *eae *variants encoding 27 different intimin types and sub-types have been established: α1, α2, β1, β2 (ξR/β2B), β3, γ1, γ2, δ (δ/β2O), ε1, ε2 (νR/ε2), ε3, ε4, ε5 (ξB), ζ, η1, η2, θ, ι1, ι2 (μR/ι2), κ, λ, μB, νB, ο, π, ρ and σ [[18-26] and unpublished data].

In HeLa and HEp-2 cells, tEPEC expresses localized adherence (LA) (with compact bacterial microcolony formation) that is mediated by the Bundle Forming Pilus (BFP), which is encoded on the EAF plasmid. In contrast, most aEPEC express the LA-like pattern, which is often detected in prolonged incubation periods (with loose microcolonies) [[2], reviewed in [3]]. However, during the characterization of an aEPEC collection, Vieira et al. [27] detected 9 strains that formed characteristic LA on HeLa cells despite the absence of BFP. Further studies showed that these strains also lacked the adhesin-encoding genes of other diarrheagenic *E. coli *pathotypes [28]. Therefore, an exemplary strain (aEPEC 1551-2) was studied in further detail. Subsequently, it was shown that in this strain the LA pattern actually corresponded to an invasion process mediated by the interaction of the intimin sub-type omicron [29]. The clinical significance of these findings in the pathogenicity of aEPEC *in vivo *is currently unknown.

Despite the fact that EPEC is generally considered an extracellular pathogen, some studies have shown limited invasion of intestinal epithelium of humans and animals by tEPEC *in vivo *[30,31]. Moreover, it has been demonstrated that some tEPEC and aEPEC strains are able to invade distinct cellular lineages *in vitro *[32-36]. Due to variations in the protocols used to determine the invasion indexes, it is difficult to compare the extent of the reported invasion ability among strains of tEPEC and aEPEC pathotypes. Furthermore, in the literature there are only a few studies on the ability of aEPEC strains to invade intestinal cells [34,35]. Most tEPEC and aEPEC invasion studies have been performed on HEp-2 [32,36,37], and polarized intestinal Caco-2 cells [33,35]. Invasion studies with aEPEC and intestinal T84 cells, which are phenotypically similar to human colon epithelial cells are still lacking. Since aEPEC is a heterogeneous pathotype [3,5,28], additional analysis of the invasive ability of aEPEC strains *in vitro *are necessary. These data could contribute to evaluate whether the invasion capacity might be considered as an additional virulence mechanism in other aEPEC strains. Therefore, in this study, we evaluated aEPEC strains expressing intimin sub-types omicron and non-omicron regarding their ability to invade HeLa and differentiated intestinal T84 cells. The eukaryotic cell structures involved in the initial steps of entry of aEPEC 1551-2 were also examined.

## Results and Discussion

Recent studies have shown that aEPEC consist of a heterogeneous group of strains, some of which could represent tEPEC strains that lost the EAF plasmid (or part of it), EHEC/STEC strains that lost *stx *phage sequences, or even *E. coli *from the normal flora that had gained the LEE region [2,27,38-40]. It remains to be elucidated whether these strains bear additional and/or specific virulence properties that are not present in tEPEC.

Recently, it has been shown that aEPEC strain 1551-2 invades HeLa cells in a process dependent on intimin omicron [29]. The aEPEC 1551-2 invasive index was about 3 folds that of tEPEC prototype strain E2348/69 tested in the same conditions. However, it is not known whether other aEPEC strains expressing intimin omicron or other intimin sub-types are also invasive. In the present study this issue was investigated.

In order to identify the intimin sub-type of four strains carrying unknown intimin sub-types, a fragment of the 3' variable region of the *eae *gene from the four aEPEC strains included in this study was amplified and sequenced (Table 1). Four different intimin types were identified: θ2 (theta), σ (sigma), τ (tau) and upsilon (Table 1). We have detected in aEPEC strains 4281-7 and 1632-7 (serotypes O104:H^- ^and O26:H^-^, respectively) two new intimin genes *eae*-τ and *eae*-ν that showed less than 95% nucleotide sequence identity with existing intimin genes. Furthermore, a third new variant of the *eae *gene (theta 2 - θ2) was identified in the aEPEC strain 1871-1 (serotype O34:H^-^). The complete nucleotide sequences of the new *eae*-θ2 (FM872418), *eae*-τ (tau) (FM872416) and *eae*-upsilon; (FM872417) variant genes were determined. By using CLUSTAL W [41] for optimal sequence alignment, we determined the genetic relationship of the three new intimin genes and the remaining 27 *eae *variants. A genetic identity of 90% was calculated between the new *eae*-τ (tau) variant and *eae-γ*2 (gama2), *eae*-θ (theta) and *eae*-σ (sigma) genes. The *eae*-upsilon; showed a 94% of identity with *eae*-ι1. The eae-θ2 (theta-2) gene is very similar (99%) to *eae*-θ of Tarr & Whittam [20] and to *eae*-γ2 of Oswald et al. [19].

**Table 1:** Characteristics of the aEPEC strains studied.

Strain	Serotype	Intimin Type	Adherence pattern	FAS test	
				
				HeLa cells	T84 cells
0621-6	ONT:H^-^	σ *	LA	+	+
1551-2	ONT:H^-^	ο	LA	+	+
1632-7	O26:H^-^	upsilon; **	DA	+	+
1871-1	O34:H^-^	θ2 **	LAL	+	+
4051-6	O104:H2	ο	AA	+	+
4281-7	O104:H^-^	τ**	LAL	+	+
E2348/69	O127:H6	α1	LA	+	+

Adhesion pattern detected on HeLa cells: localized adherence (LA), localized adherence like (LAL), aggregative adherence (AA) and diffuse adherence (DA) (Vieira et al., 2001). (*) Strains that had *eae *gene sequenced in this study and (**) strains that carry new intimin subtypes (GenBank accession numbers: 1871-1 (FM872418); 4281-7 (FM872416) and 1632-7 (FM872417).

**Figure 1:**
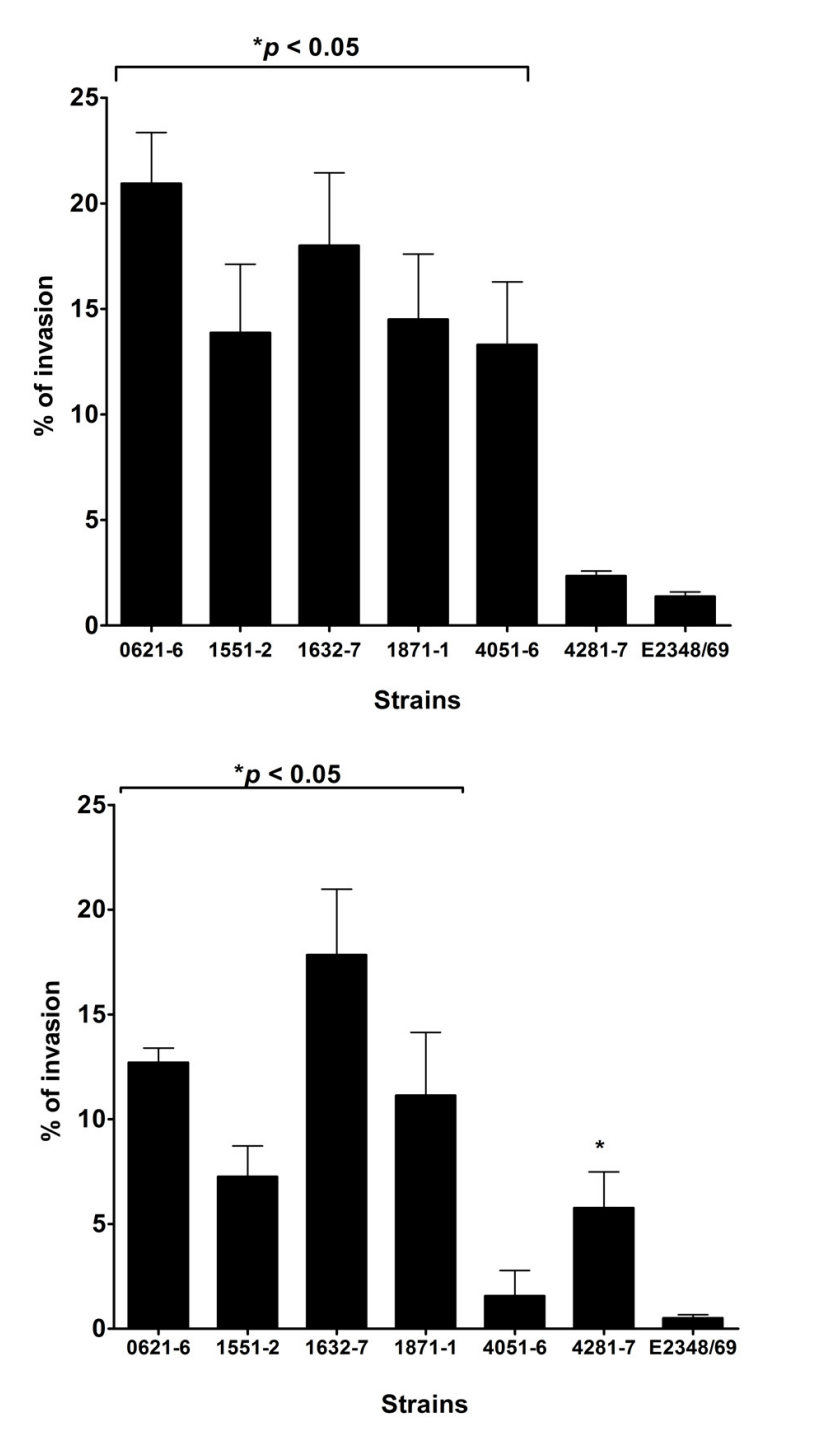
**Invasion of epithelial cells by aEPEC and tEPEC strains**. A) Percent of invasion in HeLa cells. B) Percent of invasion in T84 cells. Monolayers were infected for 6 h (aEPEC) and 3 h (tEPEC). Results of percent invasion are expressed as the percentage of cell associated bacteria that resisted killing by gentamicin and are the means ± standard error from at least three independent experiments in duplicate wells. *significantly more invasive than prototype tEPEC E2348/69 (*P *< 0.05 by an unpaired, two-tailed *t *test).

Quantitative assessment of bacterial invasion was performed with all strains, but different incubation-periods were used to test aEPEC strains (6 h) and tEPEC E2348/69 (3 h), because the latter colonizes more efficiently (establishes the LA pattern in 3 h) than aEPEC strains [3] and induce cell-detachment after 6 h of incubation (not shown). The quantitative gentamicin protection assay confirmed the invasive ability of aEPEC 1551-2 in HeLa cells and showed that 4 of the other 5 aEPEC strains studied were also significantly more invasive than tEPEC E2348/69 (Fig. 1A). The percentages of invasion found varied between 13.3% (SE ± 3.0) and 20.9% (SE ± 2.4), respectively, for aEPEC strains 4051-6 (intimin omicron) and 0621-6 (intimin sigma). When compared to tEPEC E2348/69 (intimin alpha 1) (1.4% ± 0.3), the invasion indexes of all strains were significantly higher (*p *< 0.05), except for aEPEC strain 4281-7 (intimin tau, 2.4% ± 0.3). These data confirmed that invasion of HeLa cells is not exclusively found in strains expressing intimin sub-type omicron. However, different degrees of cell invasion were observed (including strains expressing intimin omicron). Although all aEPEC strains studied were devoid of known *E. coli *genes supporting invasion [27], they are heterogeneous regarding the presence of additional virulence genes [5]. However, it remains to be evaluated whether the invasion ability as shown for aEPEC 1551-2 [29] of other aEPEC strains could be associated with the intimin sub-type. Furthermore, differences in invasion index could also be related to the presence of other factors, such as LEE and non-LEE effector proteins or expression of additional virulence genes. Alternatively, the affinity of both intimin and a specific Tir counterpart could influence the degree of manipulation of the cytoskeleton thus favoring less or more pronounced invasion.

**Figure 2:**
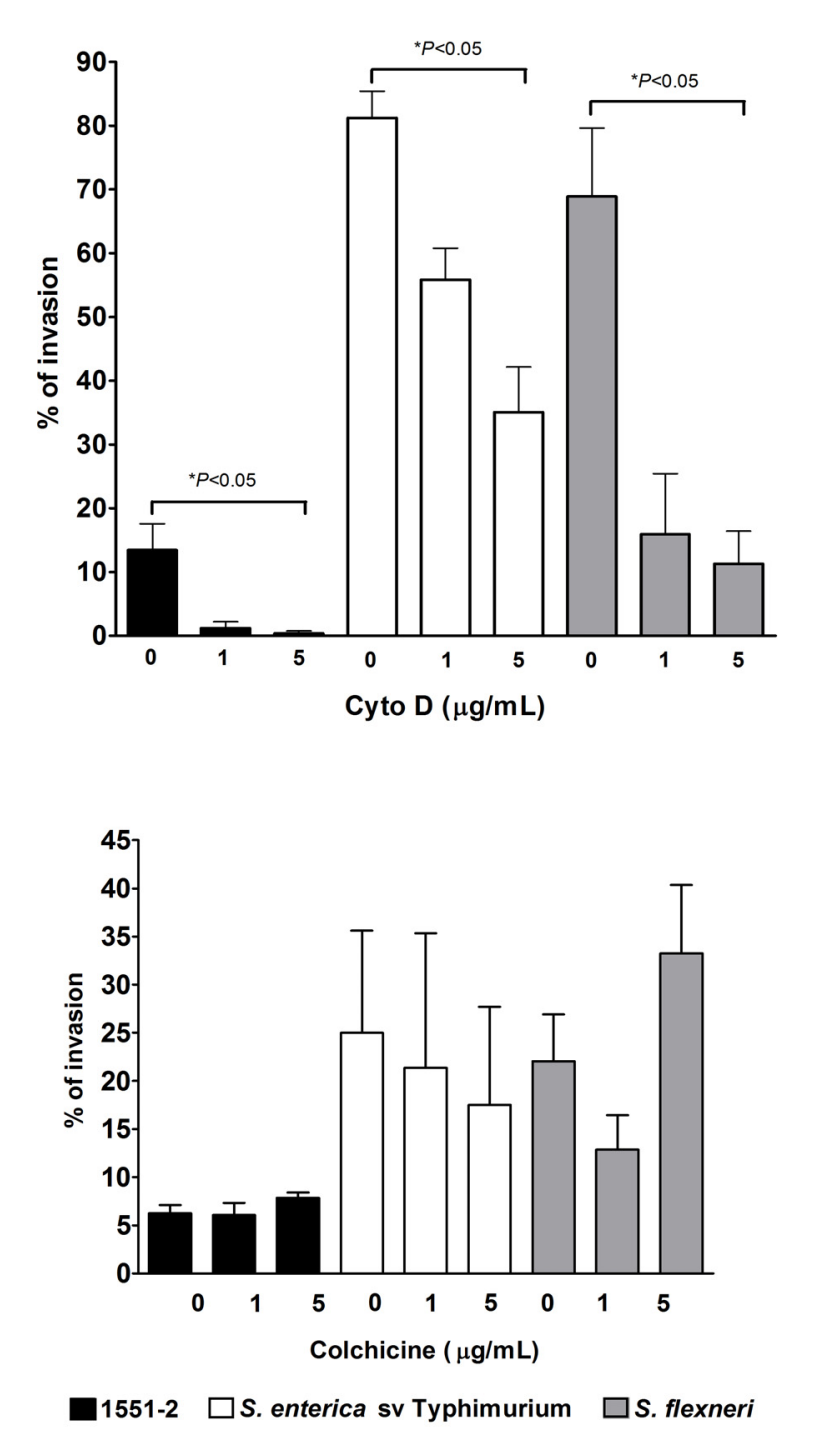
**Invasion of HeLa (epithelial) cells by aEPEC 1551-2 after treatment with cytoskeleton polymerization inhibitors**. A) Cytochalasin D; B) Colchicine. Monolayers were infected for 6 h (aEPEC) and 3 h (tEPEC). *S. enterica *sv Typhimurium and *S. flexneri *were used as controls and monolayers were infected for 4 h and 6 h, respectively. Results as percent invasion are means ± standard error from at least three independent experiments performed in duplicate. * *P *< 0.05 by an unpaired, two-tailed *t *test.

In order to identify the host cell structures and processes that might be involved in HeLa cells invasion by aEPEC 1551-2, we treated the cells with reagents affecting the cytoskeleton such as cytochalasin D (to disrupt actin microfilament formation) or colchicine (to inhibit microtubule function) prior to infection. Optical microscopy analysis revealed that treatment with cytochalasin D did not affect bacterial adhesion (data not shown). However, significantly decreased invasion by aEPEC 1551-2 (from 13.4% ± 4.1 to 1.2% ± 1.0 and 0.4% ± 0.3) was detected, as observed with the invasive *S. enterica *sv Typhimurium control strain (from 81.3% ± 4.2 to 55.9% ± 4.9 and 35.1% ± 7.1) and *S. flexneri *(from 68.9 ± 10.7 to 15.9 ± 9.5 and 11.2 ± 5.1). These results indicate that a functional host cell actin cytoskeleton is necessary for aEPEC 1551-2 uptake (Fig. 2A). In addition, this suggests that A/E lesion formation may be necessary for the invasion process since inhibition of actin polymerization resulted in both prevention of A/E lesion formation and decreased invasion. In contrast, aEPEC 1551-2 adherence (not shown) and invasion (Fig. 2B) were unaffected by colchicine cell treatment (invasion indexes of 6.2% ± 0.9 and 7.8% ± 0.6, non-treated and treated, respectively). This indicates that the microtubule network is not involved in the invasion process. As expected, *S. enterica *sv Typhimurium (25.0% ± 10.6 and 17.5% ± 10.2, respectively), and *S. flexneri *(22.1% ± 4.0 and 33.2% ± 7.1, respectively), were neither affected by treating cells with colchicine.

**Figure 3:**
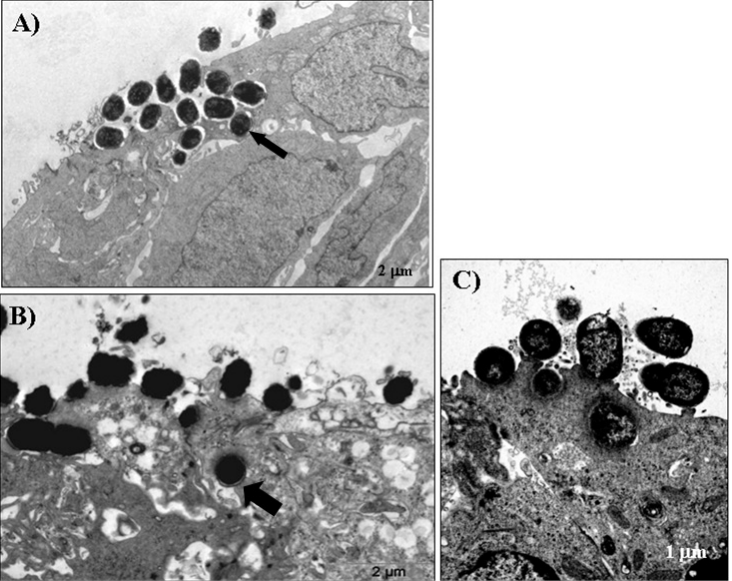
**Transmission electron microscopy of infected polarized and differentiated T84**. A) aEPEC 1551-2, B) aEPEC 0621-6 and C) prototype tEPEC E2348/69. Monolayers were infected for 6 h (aEPEC) and 3 h (tEPEC). aEPEC 1551-2 and 0621-6 were selected because, according to the data in Fig. 1B, they presented an average invasion index as compared to the other strains studied. Arrows indicate bacterial-containing vacuoles.

HeLa cells are derived from a human uterine cervix carcinoma. They are widely used to study bacterial interactions with epithelial cells yet they do not represent an adequate host cell type to mimic human gastrointestinal infections. To examine whether aEPEC strains would also invade intestinal epithelial cells, we infected T84 cells (derived from a colonic adenocarcinoma), cultivated for 14 days for polarization and differentiation, with all 6 aEPEC strains. The ability of these strains to promote A/E lesions in T84 cells was confirmed by FAS (Table 1). In the gentamicin protection assays performed with these cells, 5 of 6 strains were significantly more invasive than the prototype tEPEC strain E2348/69 (Fig. 1B). The exception was aEPEC 4051-6 (1.5% ± 1.2) that showed similar invasion index as tEPEC E2348/69 (0.5% ± 0.2). The invasion indexes of the 5 aEPEC strains varied from 5.8% ± 1.7 (aEPEC 4281-7) to 17.8% ± 3.1 (aEPEC 1632-7). These results demonstrate that besides invading HeLa cells, aEPEC strains carrying distinct intimin subtypes invade epithelial cells of human intestinal origin to different levels. Interestingly, the aEPEC invasion indexes were significantly higher than that of tEPEC E2348/69, but this comparison should be made with caution since the incubation-periods used were different. Nonetheless, it has already been demonstrated that tEPEC is unable to efficiently invade fully differentiated intestinal epithelial cells [42]. To confirm invasiveness, we examined T84 cells infected with aEPEC strains by transmission electron microscopy (TEM). This approach confirmed that 5 out of 6 aEPEC strains tested promoted A/E lesion formation and were also internalized (Fig. 3A and 3B). Under the conditions used, although some tEPEC E2348/69 cells were intra-cellular, most remained extra-cellular and intimately attached to the epithelial cell surface (Fig. 3C). Except for aEPEC strains 4281-7 in HeLa cells and 4051-6 in T84 cells, the remaining four strains tested were more invasive than tEPEC E2348/69 and showed heterogeneous invasion index in both HeLa and T84 cells.

It has been reported that the interaction between Afa/Dr adhesins, expressed by strains of the diarrheagenic *E. coli *pathotype diffusely adherent *E. coli *(DAEC), and α5β1 integrins also results in bacterial internalization [43]. Adaptation to the intracellular environment help bacteria to avoid physical stresses (such as low pH or flow of mucosal secretions or blood) and many other host defense mechanisms including cellular exfoliation, complement deposition, antibody opsonization and subsequent recognition by macrophages or cytotoxic T cells [44]. Thus, the development of mechanisms for host cell invasion, host immune response escape, intracellular replication and/or dissemination to the neighboring cells is an important strategy for intracellular bacteria [44].

**Figure 4:**
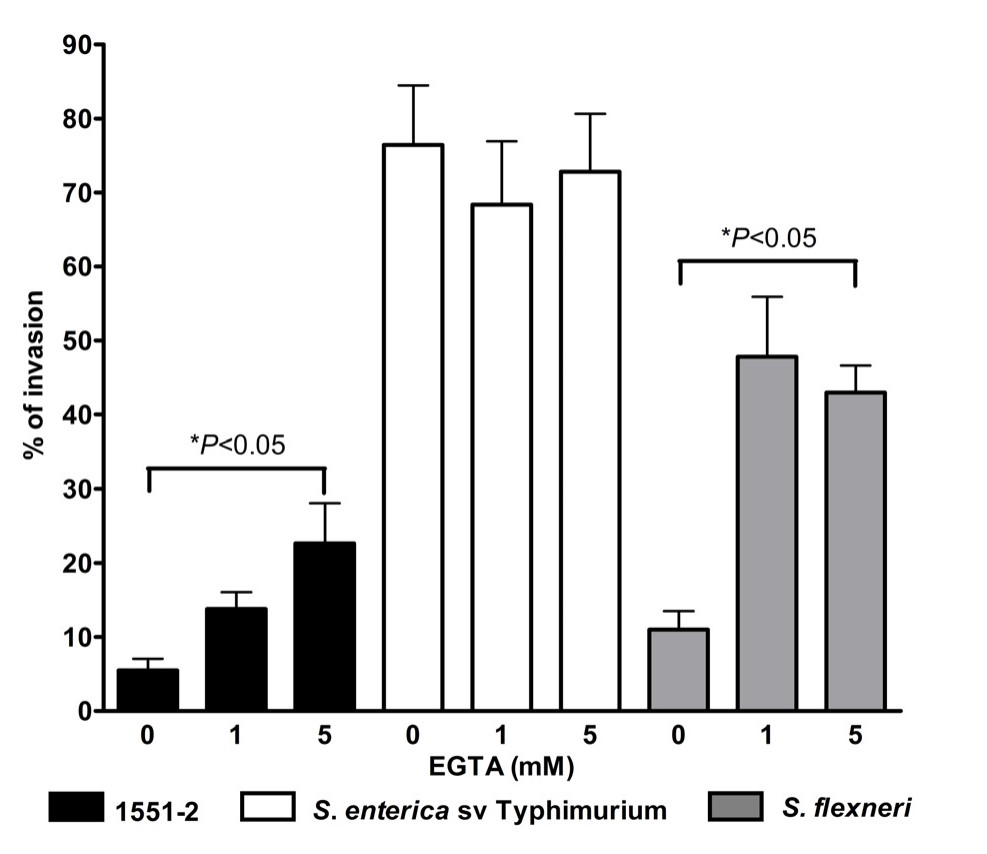
**Invasion of differentiated T84 cells by aEPEC 1551-2 after tight junction disruption by EGTA treatment**. Monolayers were infected for 6 h (aEPEC) and 3 h (tEPEC). *S. enterica *sv Typhimurium and *S. flexneri *were used as controls and monolayers were infected for 4 h and 6 h, respectively. Results of percent invasion are the means ± standard error from at least three independent experiments performed in duplicate. * *P *< 0.05 by an unpaired, two-tailed *t *test.

Tight junctions of polarized intestinal cells usually represent a barrier to bacterial invasion. Some studies have shown increased invasion indexes when cells are treated prior to infection with chemical agents that disrupt tight junctions and expose receptors on the basolateral side [35,45]. Similar observations have been made with bacteria infecting undifferentiated (non-polarized) eukaryotic cells [35,45]. These studies have shown a relationship between the differentiation stage of the particular host cells and the establishment of invasion [35,42,45]. Therefore, in order to examine whether aEPEC strains could also invade via the basolateral side of differentiated T84 cells, these cells were treated with different EGTA concentrations to open the epithelial tight junctions. The EGTA effect was accessed by optical microscopy (data not shown). Following this procedure, cells were infected with aEPEC 1551-2 and tEPEC E2348/69. Infections with *S. enterica *sv Typhimurium and *S. flexneri *were used as controls. This treatment promoted a significant enhancement of aEPEC 1551-2 and *S. flexneri *invasion, (Fig. 4) but *S. enterica *sv Typhimurium and tEPEC E2348/69 invasion indexes were not affected by the disruption of the epithelial cell tight junctions as was also reported previously [45].

**Figure 5:**
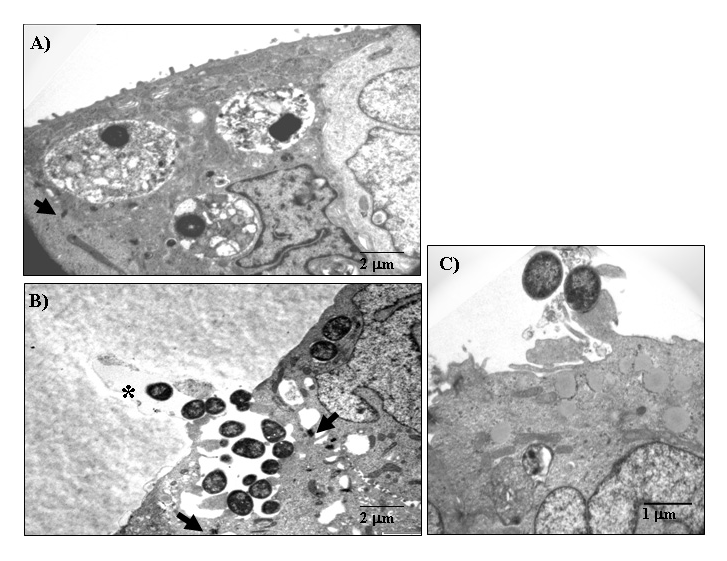
**Transmission electron microscopy of polarized and differentiated T84 cells infected via the basolateral side**. A) aEPEC 1551-2. B) aEPEC 0621-6. C) prototype tEPEC E2348/69. Monolayers were infected for 6 h (aEPEC) and 3 h (tEPEC). Arrows indicate tight junction and (*) indicates a Transwell membrane pore.

To address a putative effect of EGTA on the invasion ability of the aEPEC strains we also cultivated T84 cells for 14 days on the lower surface of a Transwell membrane. In this manner, bacterial contact with the basolateral cell surface can be achieved without prior treatment of the T84 cells. Preparations were examined by TEM and the images suggest enhanced bacterial invasion and show bacteria within vacuoles (Fig. 5) confirming the results obtained with EGTA treated T84 cells. Regarding tEPEC E2348/69, no internalized bacteria was found in the microscope fields observed. Enteropathogens may gain access to basolateral receptors and promote host cell invasion *in vivo *by transcytosis through M cells [46]. Alternatively, some infectious processes can cause perturbations in the intestinal epithelium, e.g., neutrophil migration during intestinal inflammation; as a consequence, a transitory destabilization in the epithelial barrier is promoted exposing the basolateral side and allowing bacterial invasion [47]. With regard to tEPEC, it has been reported that an effector molecule, EspF is involved in tight junction disruption and redistribution of occludin with ensuing increased permeability of T84 monolayers [48,49]. Whether EspF is involved in the invasion ability of the aEPEC strains studied *in vivo *remains to be investigated.

In conclusion, we showed that aEPEC strains expressing distinct intimin sub-types are able to invade both HeLa and differentiated T84 cells. At least for the invasive aEPEC 1551-2 strain, HeLa cell invasion requires actin filaments but does not involve microtubules. In differentiated T84 cells, disruption of tight junctions increases the invasion capacity of aEPEC 1551-2. This observation could be significant in infantile diarrhea since in newborns and children the gastrointestinal epithelial barrier might not be fully developed [45]. As observed in uropathogenic *E. coli *[50], besides representing a mechanism of escape from the host immune response, invasion could also be a strategy for the establishment of persistent disease. It is possible, that the previously reported association of aEPEC with prolonged diarrhea [8] is the result of limited invasion processes. However, the *in vivo *relevance of our *in vitro *observations remains to be established. Moreover, further analyses of the fate of the intracellular bacteria such as persistence, multiplication and spreading to neighboring cells are necessary.

## Conclusion

In this study we verified that aEPEC strains, carrying distinct intimin sub-types, including three new ones, may invade eukaryotic cells *in vitro*. HeLa cells seem to be more susceptible to aEPEC invasion than differentiated and polarized T84 cells, probably due to the absence of tight junctions in the former cell type. We also showed that actin microfilaments are required for efficient invasion of aEPEC strain 1551-2 thus suggesting that A/E lesion formation is an initial step for the invasion process of HeLa cells, while microtubules are not involved in such phenomenon. Our results also showed that tight junctions' disruption increased significantly the invasion of T84 cells by aEPEC strain 1551-2. Altogether, our findings suggest that aEPEC strains may invade intestinal cells *in vitro *with varying efficiencies and that the invasion process proceeds apparently independently of the intimin sub-type.

## Methods

### Bacterial strains and cell culture conditions

Six aEPEC strains (two carrying intimin subtype omicron and four carrying unknown intimin sub-types randomically chosen from our collection) isolated from children with diarrhea and potentially enteropathogenic due to a positive FAS assay (Table 1), and the prototype tEPEC strain E2348/69 were studied. Strains were cultured statically in Luria Bertani broth for 18 h at 37°C. Under this condition cultures reached an OD_600 _of 0.5–0.6. *Salmonella enterica *serovar Typhimurium (a gift from J.R.C. Andrade, Universidade do Estado do Rio de Janeiro) and *Shigella flexneri *M90T [51] were used as controls in some experiments in infection assays of 4 and 6 h, respectively. All strains were shown to be susceptible to 100 μg/mL of gentamicin prior to the invasion experiments. HeLa cells (10^5 ^cells) were cultured in Dulbecco Modified Eagle Medium (DMEM) supplemented with 10% bovine fetal serum (Gibco Invitrogen) and 1% antibiotics (Gibco Invitrogen), and kept for 48 h at 37°C and 5% CO_2_. T84 cells (10^5 ^cells) were cultured in DMEM-F12 medium (Gibco Invitrogen) supplemented with 10% bovine fetal serum (Gibco Invitrogen), 1% non-essential amino acids (Gibco Invitrogen) and 1% antibiotics (Gibco Invitrogen), and kept for 14 days at 37°C and 5% CO_2 _for differentiation. For some transmission electron microscopy analysis, T84 cells (10^5 ^cells) were cultivated on the lower surface of Corning Transwell polycarbonate membrane inserts pore size 3.0 μm, membrane diameter 12 mm. In addition to apical adhesion this procedure allowed bacterial inoculation directly at the basolateral surface of the cells avoiding the use of chemical treatment to expose such surface.

### Serotyping

The determination of O and H antigens was carried out by the method described by Guinée et al. [52] employing all available O (O1-O185) and H (H1-H56) antisera. All antisera were obtained and absorbed with the corresponding cross-reacting antigens to remove the nonspecific agglutinins. The O antisera were produced in the Laboratorio de Referencia de *E. coli *(LREC) (Lugo, Spain) and the H antisera were obtained from the Statens Serum Institut (Copenhagen, Denmark).

### Typing of intimin (*eae*) genes

Intimin typing was performed by sequencing a fragment of the 1,125 bp from 3' variable region of the *eae *genes from four aEPEC strains included in this study. The complete nucleotide sequences of the new θ2 (FM872418), τ (FM872416) and ν (FM872417) variant genes were determined. The nucleotide sequence of the amplification products purified with a QIAquick DNA purification kit (Qiagen) was determined by the dideoxynucleotide triphosphate chain-termination method of Sanger, with the BigDye Terminator v3.1 Cycle Sequencing Kit and an ABI 3100 Genetic Analyzer (Applied Bio-Systems). The new *eae *sequences of strains analyzed were deposited in the European Bioinformatics Institute (EMBL Nucleotide Sequence Database).

### Quantitative invasion assay

Quantitative assessment of bacterial invasion was performed as described previously [53] with modifications. Briefly, washed HeLa and polarized and differentiated T84 cells were infected with 10^7 ^colony-forming units (c.f.u.) of each aEPEC strain for 6 h or 3 h for tEPEC E2348/69. The different incubation-periods used were due to the more efficient colonization of tEPEC in comparison with the aEPEC strains; moreover, tEPEC E2348/69 induced cell-detachment in 6 h. Thereafter, cell monolayers were washed five times with PBS, and lysed in 1% Triton X-100 for 30 min at 37°C. Following cell lysis, bacteria were re-suspended in PBS and quantified by plating serial dilutions onto MacConkey agar plates to obtain the total number of cell-associated bacteria (TB). To obtain the number of intracellular bacteria (IB), a second set of infected wells was washed five times and further incubated in fresh media with 100 μg/mL of gentamicin for one hour. Following this incubation period, cells were washed five times, lysed with 1% Triton X-100 and re-suspended in PBS for quantification by plating serial dilutions. The invasion indexes were calculated as the percentage of the total number of cell-associated bacteria (TB) that was located in the intracellular compartment (IB) after 6 h (or 3 h for tEPEC E2348/69) (IBx100/TB) of infection. Assays were carried out in duplicate, and the results from at least three independent experiments were expressed as the percentage of invasion (mean ± standard error).

### Cytoskeleton polymerization inhibitor

In order to evaluate the participation of cytoskeleton components in the invasion of aEPEC 1551-2, HeLa cell monolayers were incubated with 1 and 5 μg/mL of Cytochalasin-D or Colchicine (Sigma-Aldrich, St. Louis, MO) 60 min prior to bacterial inoculation [33]. After that, cells were washed three times with PBS and the invasion assay was performed as described above. *S. enterica *sv Typhimurium and *S. flexneri *were used as controls.

### EGTA treatment for tight junction disruption

In order to evaluate the interaction of aEPEC 1551-2 with the basolateral surfaces of T84 cells, differentiated cell monolayers (14 days) were incubated with 1 or 5 mM of EGTA (Sigma-Aldrich, St. Louis, MO) 60 min prior to bacterial inoculation [35]. After that, cells were washed three times with PBS and the invasion assay was performed as describe above. *S. enterica *sv Typhimurium and *S. flexneri *were used as controls.

### Detection of actin aggregation

To detect actin aggregation the Fluorescence Actin Staining (FAS) assay was performed as described previously [12]. Briefly, cell monolayers were infected for 3 h, washed three times with PBS and incubated for further 3 h with fresh medium. Subsequently, monolayers were washed five times with PBS, fixed with 3.5% paraformaldehyde, and lysed in 1% Triton X-100 for 5 min at room temperature. Monolayers were then washed three times, incubated in a dark chamber with 5 μg/mL phalloidin (20 min), and washed. Coverslips were mounted in glycerol with 0.1% para-phenylenediamine to reduce bleaching.

### Transmission Electron Microscopy

T84 cells were cultured in Transwell membranes (Costar) for 14 days and infected as described above. Then they were washed 3 times (10 min each) with D-PBS (Sigma) and fixed with 2% glutaraldehyde (Serva) for 24 h at 4°C. After fixation, cells were washed 3 times with D-PBS (10 min) and post-fixed with 1% osmium tetroxide (Plano). Cells were dehydrated through a graded ethanol series (30%, 50% and 70%), then filters were cut out from the cell culture system holder and preparations were treated with ethanol (90%, 96% and 99.8%), followed by propylenoxid (100%), Epon:Propylenoxid (1:1, Serva), and Epon 100%. Afterward, filters were embedded in flat plates and kept for 2 days for polymerization. Ultrathin sections were prepared, stained with 4% uranyl acetate (Merck) and Reynold's lead citrate (Merck), and were examined with a Tecnai G2 Spirit Twin, Fei Company at 80 kV.

Alternatively, T84 cells were cultured on 35 mm diameter plates for 14 days. Infection, fixation and dehydration were performed as described above. Subsequently, the cells were examined with a LEO 906E transmission electron microscope (Zeiss, Germany) at 80 kV.

### Statistical analyses

Differences in the percentages of invasion were assessed for significance by using an unpaired, two-tailed *t *test (GraphPad Prism 4.0).

## Authors' contributions

DY and RH carried out all invasion assays and drafted this manuscript. MB, GD and AM carried out the typing of the *eae *gene. LG and SMC carried out transmission electron microscopies of T84 cell. JEB performed serotyping. MAS and JB contributed to the experimental design and co-wrote the manuscript with TATG. TATG supervised all research, was instrumental in experimental design, and wrote the final manuscript with DY.

This research was carried out as thesis work for a PhD (DY) in the Department of Microbiology at the Universidade Federal de São Paulo. All authors read and approved the final manuscript. The authors declare that they have no competing interests.
