# Correction: Invasiveness as a putative additional virulence mechanism of some atypical Enteropathogenic *Escherichia coli *strains with different uncommon intimin types

**DOI:** 10.1186/1471-2180-9-235

**Published:** 2009-11-11

**Authors:** Denise Yamamoto, Rodrigo T Hernandes, Miguel Blanco, Lilo Greune, M Alexander Schmidt, Sylvia M Carneiro, Ghizlane Dahbi, Jesús E Blanco, Azucena Mora, Jorge Blanco, Tânia AT Gomes

**Affiliations:** 1 Universidade Federal de São Paulo (UNIFESP), São Paulo, Brazil, Rua Botucatu, 862, 3° andar, Vila Clementino, São Paulo, São Paulo, CEP 04023-062, Brazil; 2 Institut für Infektiologie, Zentrum für Molekularbiologie der Entzündung, Westfälische Wilhelms-Universität, Münster, Germany; 3 E. coli Reference Laboratory (LREC), Department of Microbiology and Parasitology, Faculty of Veterinary, University of Santiago de Compostela, Lugo, Spain; 4 Laboratório de Biologia Celular do Instituto Butantan, São Paulo, Brazil

## 

We lately detected that some important errors were introduced during the production of the last version of our article [1] regarding some Greek letters used to classify intimin subtypes. We regret that these errors were introduced in the final version.

In Table 1, the Greek letters used to nominate intimin subtype omicron should be corrected (ο instead of μ). Furthermore, intimin upsilon (Greek letter- υ) appears with a wrong symbol (ν). Please, find below the corrected version of Table 1.

**Table 1:** Characteristics of the aEPEC strains studied.

Strain	Serotype	Intimin Type	Adherence pattern	FAS test	
				
				HeLa cells	T84 cells
0621-6	ONT:H^-^	σ *	LA	+	+
1551-2	ONT:H^-^	ο	LA	+	+
1632-7	O26:H^-^	υ **	DA	+	+
1871-1	O34:H^-^	θ2 **	LAL	+	+
4051-6	O104:H2	ο	AA	+	+
4281-7	O104:H^-^	τ **	LAL	+	+
E2348/69	O127:H6	α1	LA	+	+

In "Results and Discussion" (Page 3, Paragraph 1) and in "Methods" ("Typing of intimin genes", Page 8), intimin upsilon (Greek letter- υ) appears again with a wrong symbol (ν).
